# Equine Asthma Diagnostics: Review of Influencing Factors and Difficulties in Diagnosing Subclinical Disease

**DOI:** 10.3390/ani14233504

**Published:** 2024-12-04

**Authors:** Lioba Lendl, Ann Kristin Barton

**Affiliations:** Equine Clinic Hochmoor, Ruthmannstr. 10, 48712 Gescher, Germany

**Keywords:** horse, chronic respiratory disease, inflammatory airway disease

## Abstract

This literature review focuses on diagnostics of equine asthma (EA), possible influencing factors on diagnostic techniques and latest developments in diagnosing horses during EA remission or with subclinical disease. Routine EA diagnostics include a clinical examination of the respiratory system with percussion and auscultation including a rebreathing examination, and clinical pathology including white blood cells and arterial blood gas analysis. Subsequent diagnostics include bronchoscopy to evaluate the amount and viscosity of respiratory secretion, bronchoalveolar lavage, and the cytology of tracheal aspirates (TAs) and bronchoalveolar lavage fluid (BALF). The grading of EA severity is built on respiratory effort at rest, which is increased in severe equine asthma. The inflammatory subtype is based on BALF cytology, while TA cytology helps to rule out previous bacterial infections. Different factors have an impact on the airways regarding the structure of the epithelium, cytology, and inflammatory markers possibly influencing the diagnosis of EA. Short-term exercise increases the total cell count and inflammatory mediators identified in the BALF of human patients, asymptomatic horses, and other species. Other factors involve cold or chlorinated air, long-term training effects, and concurrent additional respiratory disease, in particular exercise-induced pulmonary hemorrhage. As BALF cytology may be unremarkable during EA remission and low-grade disease, exercise tests and other factors stressing the bronchial epithelium may help to diagnose these patients.

## Simple Summary

Equine asthma is a common pulmonary disorder among equine patients. Up to seventy percent of young sports horses are affected, although they do not show clinical signs like coughing. The diagnosis of equine asthma in these horses is challenging but important, as mild-to-moderate equine asthma may deteriorate with time. This review summarizes options to diagnose equine asthma early in the disease process and discusses possible difficulties. Interestingly, stressing the airways using exercise or cold air may help to identify horses suffering from mild disease or severe equine asthma in remission.

## 1. Introduction

Equine asthma is mainly a neutrophilic inflammatory disease of the lower airways in horses. It is characterized by mass hypertrophy of the smooth bronchial musculature and the obstruction resulting from this, as well as hyperplasia of the goblet cells and dyscrinia. Currently, the roles and types of hypersensitivity reactions to environmental antigens remains a topic of debate in the scientific community [1,2,3].

While over two hundred endotypes are known in human asthma [4], only two phenotypes have been defined in horses: a mild-to-moderate (MEA) and a severe form (SEA). MEA, formerly known as inflammatory airway disease (IAD), is more frequently diagnosed in horses under the age of seven, while SEA, formerly known as recurrent airway obstruction (RAO) or heaves, is more prevalent in older horses. SEA is a chronic, progressive disease; therefore, owners frequently report recurrent clinical signs or episodes lasting for at least four weeks or longer. On the contrary, the majority of horses with MEA appear to recover [2]. Coughing and a slight decline in performance may be the only clinical signs of MEA. MEA has been reported to have a high prevalence of up to 70% [5,6,7]. A prevalence of up to 14% is documented globally for SEA [8]. Disease exacerbation in horses with SEA is characterized by an elevated respiratory rate, dyspnea at rest, and abdominal breathing resulting from bronchospasm of the bronchial muscles. The clinical signs of horses suffering from SEA are typically more severe during the winter months, due to the presence of specific triggers including hay, bedding, and dust. In contrast, horses suffering from severe equine pasture asthma (EPA), which is characterized by reversible airway obstruction triggered by grazing on pasture, demonstrate more severe symptoms during the summer months [1].

There is evidence suggesting that the severe form of equine asthma can evolve from the mild-to-moderate form [1,9]. It is recommended that a diagnosis is achieved early on in the disease process, as horses suffering from MEA already demonstrate remodeling in the form of epithelial hyperplasia, a thickened lamina propria, and smooth muscle fibrosis [10]. The remodeling of the bronchial smooth muscle, the extracellular matrix of the lamina propria, and the pulmonary arteries in horses suffering from SEA progresses during the course of the disease. In particular, remodeling of the bronchial smooth muscle is not fully reversible. Even in SEA remission, an increase in airway smooth muscle (ASM) of 50% compared to healthy horses was shown [11]. On the other hand, remodeling of the extracellular matrix and pulmonary arteries appears to be largely reversible after environmental improvement and inhalation therapy [12,13]. Therefore, if MEA is diagnosed at an early stage, the possibility of recovery exists, provided that irreversible remodeling has not yet occurred. EA with irreversible remodeling is not curable and can therefore only be managed effectively through good management.

## 2. Diagnosis of EA

### 2.1. Clinical Examination and Laboratory Tests

Although multiple techniques involving laboratory tests and biomarkers have been evaluated regarding their efficacy in diagnosing equine asthma, the history of recurrent expiratory dyspnea and a detailed clinical examination of the respiratory system during exacerbation remains the basis of diagnosing equine asthma. In addition, venous blood taken for the evaluation of leukocytes gives information about concurrent respiratory infection. Arterial blood gas analysis (PaO2, PaCO2, AaDO2) allows the severity of gas exchange impairment to be graded. Unfortunately, these basic diagnostic tests are often unremarkable during SEA remission or in MEA. Poor performance can also be caused by musculoskeletal abnormalities or upper airway disease and is often multifactorial [2].

### 2.2. Bronchoscopy

Endoscopy of the upper airways is a valuable tool for the diagnosis of upper respiratory tract diseases affecting the larynx, pharynx, and guttural pouches. These conditions have the potential to cause obstruction and therefore respiratory noise and may also impair performance [1].

Bronchoscopy allows for the assessment of the quantity and viscosity of tracheal and bronchial secretions [14]. The quantity of mucus is significantly correlated with the neutrophil count in tracheal aspirates (TAs) and bronchoalveolar lavage fluid (BALF) cytology. While there is considerable interobserver variance in grading mucus viscosity, color, and location, mucus volume within the trachea has a high interobserver agreement [14]. Although mucus accumulation is a common finding in respiratory disease, it is not necessarily an indicator of EA. A minimal amount of mucus (120 mL/day) was also found in healthy horses within the trachea [7,14]. Tracheal aspirates can either be taken through the endoscopic working channel or as a transcutaneous tracheal aspirate. Although the latter technique is no longer commonly performed, transcutaneous tracheal aspiration or the wash technique offers the advantage of reduced pharyngeal contamination by bypassing the upper airways [15].

It may not be possible to differentiate between bacterial infections and equine asthma when assessing a sample macroscopically. Therefore, cytology of the tracheal aspirate is evaluated through microscopic examination following Diff-Quick staining [16]. A diagnosis of airway inflammation should be made if TA cytology reveals a neutrophil count of greater than 20%. The presence of intracellular bacteria, phagocytosed by macrophages and neutrophil granulocytes, indicates an infectious disease in contrast to a pure inflammatory airway disease. In case of doubt, the TA should be cultured for aerobic and anaerobic bacteria. Additionally, the sample may be contaminated with bacteria from the oropharynx, which appear extracellular in cytology, or fungal hyphae and pollen [15]. As the cells from the lungs are transported along the trachea, a sample from the trachea contains older and more incomplete sets of cells compared to what would be detected in the lungs [14]. For this reason, BALF cytology is a more sensitive method for diagnosing EA, especially MEA [17,18,19].

In general, BALF cytology is a more useful diagnostic tool for diffuse lung disease, such as EA, whereas TA cytology represents the composition of the trachea and may originate from any location in the lower airways by mucociliary transport. This can therefore reflect focal or diffuse lung disease. Several studies found no correlation between cytology results comparing TA and BALF in horses with chronic lung disease [20,21]. One study found a correlation between neutrophil percentages in TA and BALF cytology [17]. Nevertheless, TA is an ineffective method for diagnosing mild-to-moderate equine asthma, mainly because of the different subtypes and the possibility of there being no mucus in the trachea. TA cytology does not permit a diagnosis of eosinophilic or mastocytic subtypes to be made, as these cells are not commonly found in TA. Eosinophil and mast cell ratios in BALF are a better representation of the status of the lower airways [19,21]. It is important to note that acute respiratory distress and infectious lung diseases are contraindications for bronchoalveolar lavage (BAL).

Bronchoalveolar lavage (BAL) can be performed under endoscopic control or blindly with a BAL catheter [15]. Both methods are commonly used as described elsewhere [17,20]. A volume of 250–500 mL of saline is recommended for this purpose; a minimum of 50% of the fluid should be recovered in order to obtain a sample with good diagnostic value [2,22]. It would be advisable to standardize the volume more, as the neutrophil percentages are significantly higher when a smaller volume of saline is used [23]. The volume of fluid used for BAL sampling does not exhibit a significant influence on the total cell count (TCC) and percentages of other cell types than neutrophils in BALF. The total cell count can be determined by either a hemocytometer or an automated hematology analyzer, provided that the sample is free from visible mucus [15]. In addition, the utilization of “total cell count in epithelial lining fluid” (ELF) would serve as a superior parameter for ensuring comparability. The total cell count in ELF can be calculated by measuring urea in blood plasma and BALF. Urea diffuses through numerous body compartments, including the lungs. Its ratio between peripheral blood and BALF concentration allows the effect of dilution to be calculated as follows: ELF (mL) = [total amount of urea in BALF recovered (mg)]/[concentration of urea in plasma (mg/mL)] [24]. With the recovered volume of BALF, total cell count in BALF (hemocytometer), and urea in blood plasma and BALF, it is possible to calculate the absolute cell count in ELF and thereby the total cell count in ELF in cells/μL. The physiological cell count in ELF is estimated to be below 15,500 to 21,700 cells per microliter [25]. Currently, it is uncommon to specify the total cell count in ELF, but it is more comparable than TCC in BALF.

The BALF cytology in MEA is characterized by an increase in neutrophil granulocytes to >10% and/or eosinophil granulocytes and/or mast cells to >5% [2]. Variability in both physiological and pathological values should be acknowledged. The values can be influenced by various factors, including the diagnostic technique, environmental conditions, and the population examined [1]. The abovementioned reference ranges may be imprecise in particular in sports horses, where subtle changes within the reference may go unnoticed and therefore may leave horses without diagnosing their disease [26].

In SEA exacerbation, an increase in neutrophils > 25% is characteristic of BALF cytology [2]. To date, it remains difficult to distinguish MEA and SEA when patients are in remission. While moldy hay challenge may be useful in research settings, this is hard to apply in a clinical setting due to poor owner acceptance [1]. On the other hand, it is essential to differentiate between MEA and SEA in remission to evaluate the potential treatment options and prognosis.

### 2.3. Radiography and Ultrasonography

The diagnostic value of radiography and ultrasonography is considered by the authors to be limited, especially for the diagnosis of MEA [27,28]. A correlation between radiographic findings and the severity of disease and clinical signs could be established for SEA only [29]. In these cases, radiographs are assessed for lung pattern changes, lung remodeling, and increased lung radiopacity.

Ultrasonography provides information on the lung surface. Comet-tail artefacts are able to indicate a non-specific inflammation of the lung. Endobronchial ultrasonography via endoscopy can be used to distinguish between severe asthmatics in exacerbation and healthy horses. However, this method does not allow for a diagnosis of asthmatics in remission [30].

In addition to the lack of significance for MEA diagnostics, these imaging procedures are not specific to EA, which is why these methods are only suitable for the exclusion of differential diagnoses such as pulmonary abscesses [27,31].

### 2.4. Use of Biomarkers

Multiple biomarkers have been studied with regard to their efficacy in supporting a diagnosis of EA (Table 1). While they are widely used in research to study the pathogenesis of EA, they are not commonly used for routine diagnostics in equine practice. In general, a considerable number of biomarkers are only applicable to SEA diagnostics and are frequently unable to be utilized for more specific MEA diagnostics or during SEA remission [32]. In addition, BALF biomarkers often have a higher diagnostic value than blood biomarkers, which would have the advantage of easy execution in the field, where BALF is rarely taken routinely. Unfortunately, blood biomarkers often have a low sensitivity and/or specificity [33]. Lung-specific biomarkers and non-specific inflammation markers (e.g., acute-phase proteins) have been described, commonly based on ELISA techniques [34]. Numerous biomarkers are suitable for diagnostics of severe equine asthma (Table 1). As this review focuses on the diagnostics of mild–moderate equine asthma, a short update is given on biomarkers, which may be relevant for staging EA severity or diagnosing MEA in the future, as follows:

Surfactant protein-D is a protective protein in pulmonary inflammation, which increases in serum, particularly in the context of mild and severe neutrophilic asthma. Haptoglobin is an acute-phase protein that is elevated in serum even in horses suffering from MEA [34]. The combination of the measurement of both biomarkers demonstrates a sensitivity and specificity of 100% [33]. Secretoglobin is secreted by club cells and is an anti-inflammatory protein that is reduced in the BALF of asthmatic horses [34,35]. No significant decline can be detected in the blood. However, in combination with a HOARSI score [36] greater than 2, it is a viable blood biomarker [34]. The acute-phase protein Serumamyloid A (SAA) is elevated in the blood in acute inflammatory phases of EA in exacerbation as a marker of systemic inflammation [32].

Neutrophil gelatinase-associated lipocalin (NGAL) is a biomarker for kidney disease that has been identified in human medicine. It is produced by neutrophil granulocytes and the epithelial cells of various organs, including the kidneys, intestines, liver, and lungs. The concentration of NGAL in BALF has been observed to increase in correlation with the severity of the disease, although no significant difference has been noted between the various MEA subtypes. Consequently, its diagnostic value in MEA is limited [37].

Matrix metalloproteinases (MMPs) and their tissue inhibitors (TIMPs) have been demonstrated to be useful in the diagnosis of a number of lung diseases, given that they are no longer in equilibrium during lung remodeling processes. The ratio of MMP-8 and TIMP-1 in BALF has been shown to be effective in differentiating between MEA and SEA, with the ratio increasing in SEA and decreasing in MEA and SEA in remission [38].

Procalcitonin, as a precursor protein of calcitonin, has the potential to serve as a promising blood biomarker as it is elevated in the blood serum of horses with chronic pneumopathies. The possibility of differentiating between various respiratory diseases with the aid of procalcitonin still requires further research [39].

**Table 1:** List of various biomarkers detectable either in blood serum, BALF, exhaled breath condensate, or surfactant. Some biomarkers are verifiable in SEA only; others can be found in horses with MEA as well.

Biomarker	Suitable for MEA/SEA	BALF/Blood Serum	Increase or Decrease	Literature
Surfactant protein-D	MEA + SEA	Blood serum	increase	[33,34]
Haptoglobin	MEA + SEA	Blood serum	increase	[32,33,34]
Secretoglobin	MEA + SEA	BALF, blood serum	decrease	[34,35]
SAA	SEA	Blood serum	increase	[32]
Neutrophil gelatinase-associated Lipocalin	(MEA +) SEA	BALF	increase	[37]
MMPs + TIMPs	MEA + SEA	BALF	disturbed balance	[38]
Procalcitonin	(MEA +) SEA	Blood serum	increase	[39]
NETs	SEA	BALF	increase	[40]
Myeloperoxidase	SEA, even in remission	BALF	increase	[41]
Myo-Isonitol	SEA	BALF	decrease	[42]
Formate	SEA	BALF	increase	[42]
Isopropanol	SEA	BALF	increase	[42]
Ethanol	SEA	Exhaled breath condensate	increase	[42]
Interleukin-1beta	SEA	BALF	increase	[43]
Tumor Necrosis Factor-alpha	SEA	BALF	increase	[43,44]
Interleukin-8	SEA	BALF	increase	[43,44]
Interleukin-4	SEA	BALF	increase	[43]
Interferon-gamma	SEA	BALF	increase	[43,44]
Thromboxane B2	SEA	Blood serum	increase	[45]
Prostaglandin E2	SEA	BALF	increase	[46]
Prostaglandin F	SEA	BALF	increase	[46]
Histamin	SEA, even in remission	BALF	increase	[47]
Cyclic phosphatidic acid	SEA	Surfactant	increase	[48]
Diacylglycerol	SEA	Surfactant	increase	[48]
8-hydroxy-2-deoxyguanosine	SEA	Blood serum	increase	[49]
Transferrin	SEA	BALF	decrease	[35]
IgE + IgG antibodies specific for Aspergillus fumigatus	SEA	BALF	increase	[50]

In the most recent Havemeyer workshop (2019), it was emphasized that further research is required to improve the diagnosis of mild-to-moderate EA cases. The objective should be to diagnose EA at an earlier stage, allowing for a differentiation between mild and moderate cases. Horses presenting with poor performance but without clinical signs of respiratory disease could be assigned to the mild form, whereas horses showing respiratory symptoms would fit the classification of the moderate form. How this classification would fit cytological criteria is yet to be determined. There are several scoring systems for EA [51,52], but it is quite difficult to distinguish mildly affected horses from healthy ones or from those with SEA in remission. Therefore, research focusing on the differentiation between healthy and mildly affected horses is needed [1]. It is of the utmost importance that MEA, a disease with such a high prevalence, can be diagnosed with a high degree of accuracy, which is currently not possible. For this reason, it might be beneficial to identify stress factors that possibly offer greater diagnostic sensitivity or specificity concerning the identification and classification of EA. Future studies should focus on the question of whether changes in healthy and EA-affected horses are induced by these stress factors themselves, or if these stress factors lead to significantly more changes in affected animals, so that they can be used to improve early diagnostics.

**Figure 1:**
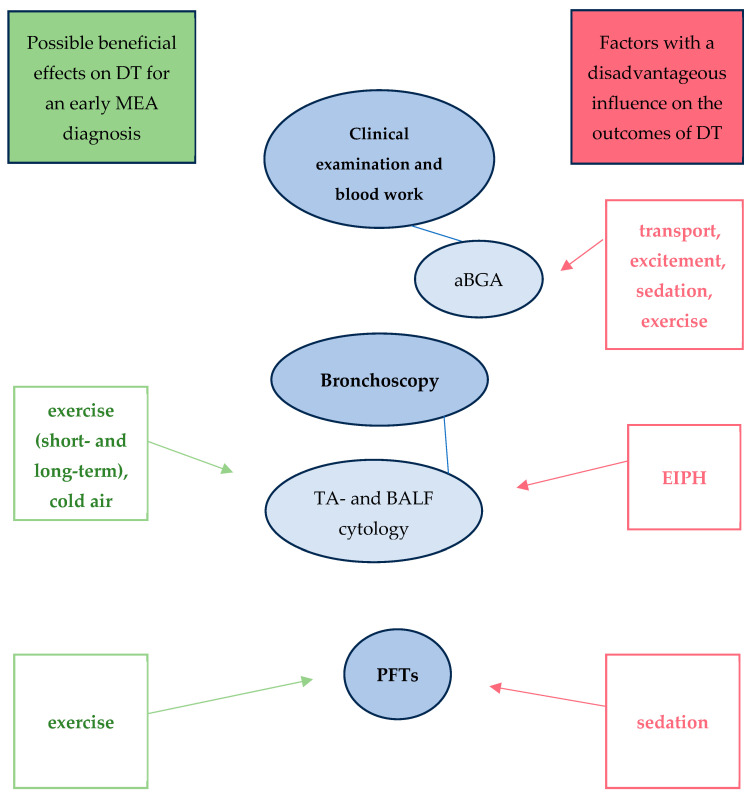
Different factors influencing the techniques used for EA diagnostics. Blue: selection of techniques used for EA diagnostics, the results of which can be affected by a number of variables. Red: factors whose influence can lead to a distortion of the results of these diagnostic techniques. Green: factors which have the potential to influence diagnostic techniques in a manner that may facilitate an earlier diagnosis of MEA. DT: diagnostic techniques, PFTs: pulmonary function tests.

Various beneficial and adverse factors influence common diagnostic tests (Figure 1). Therefore, it is important to be aware of these and to combine several procedures to increase the diagnostic accuracy.

## 3. Effects of Sedation and Exercise on aBGA

Arterial blood gas analysis (aBGA) is an effective method for assessing alveolar ventilation and thus gas exchange. However, it is not a reliable diagnostic tool for horses with MEA or EA in remission [53]. In horses with SEA, the most significant value is decreased PaO2. Some horses also exhibit increased PaCO2 and low pH values [54].

The impact of sedation on aBGA has been reported [55]. The aBGA should be carried out prior to sedation for bronchoscopy, because sedation has a negative effect on the values in healthy and EA-affected horses. Sedation with detomidine and butorphanol results in a decrease in PaO2 and PvO2 and an increase in PaCO2 and P(A-a)O2 due to hypoventilation, and therefore results in an imbalance of ventilation and perfusion [56]. Furthermore, a decrease in heart rate is observed under sedation. While the respiratory rate remains largely unaltered following sedation with detomidine, the addition of butorphanol results in a notable decline. The interplay between pulmonary and cardiovascular effects ultimately leads to impaired gas exchange. Among sedation regimens involving romifidine and butorphanol, the combination of these agents demonstrates more pronounced negative effects on oxygen saturation than the combination of romifidine, butorphanol, and acepromacin [57].

Exercise is another factor which has a negative impact on aBGA, especially PaO2, due to alveolar hypoventilation from augmented pulmonary resistance and increased work of breathing during exercise. The values of pO2, pCO2, and pO2(A-a) taken after exercise are highly variable, depending on the training status and condition of the horse [58]. Therefore, post-exercise aBGA is not a suitable tool to improve EA diagnostics, as even healthy horses exhibit exercise-induced hypoxemia [59]. Furthermore, no correlation was identified between the aBGA results of MEA-affected horses after exercise and the cytological MEA subtypes [60]. Consequently, the blood sample for aBGA should not only be obtained prior to sedation, but also prior to the exercise test. Furthermore, the effect of transportation to the clinic, for instance, must be taken into account. In addition to alterations in immune function, which can be discerned during both long-distance and short-term transport, the activation of the hypothalamic–pituitary–adrenal (HPA) axis during short-term transportation indicates that the horse is experiencing a stressful situation [61]. An increase in respiratory rate and a reduction in the depth of breathing result in hypoventilation of the lungs, leading to an imbalance between ventilation and perfusion, which impairs gas exchange. It is recommended that the horses be kept in a stable for a few minutes/hours after arrival to allow for a period of calm before measuring arterial blood gases [62,63].

## 4. Effect of Exercise and Sedation on Pulmonary Function Tests

Pulmonary function testing (PFT) is a valuable method for determining the extent of lung function impairment [64]. This technique is currently limited due to its challenging and expensive implementation.

The gold standard for measuring airflow and changes in pleural pressure in horses is esophageal balloon pneumotachography. This method is invasive, as it requires the insertion of a balloon catheter into the esophagus, which is then connected to a pressure transducer. This method is mainly utilized in research to examine pulmonary resistance, elastance, pleural pressure, and compliance [65].

In human medicine, the gold standard is spirometry [66]. This gives information on respiratory rate, peak inspiratory and expiratory flows, tidal volume, time to peak flow, and forced vital capacity. However, the implementation of these tests in horses is considerably more complex than in humans. Horses are required to wear face masks to measure airflow on a breath-by-breath basis. These masks must be available in a range of sizes to accommodate the varying dimensions of horses. Some horses might have difficulties in accepting the procedure. In the future, test protocols will have to be developed for horses. It is important to note that it is challenging to achieve a timed, forced, and complete inspiration and expiration in horses [64]. The results of the spirometry tests conducted with spontaneous breathing at rest indicate that there are no significant differences between healthy and MEA-affected horses [18]. In contrast, deviations from physiological values become more obvious during exercise. Horses suffering from bronchitis have a decreased ratio of time for expiration to time for inspiration in comparison to healthy ones during exercise. There is a significant correlation between the spirometry measurement results and the percentage of neutrophils in the TAs [67]. This indicates that spirometry after exercise provides valuable information about the impairment of lung function due to EA. In addition, it is feasible to perform a single forced exhalation in sedated horses [18]. However, this is challenging to execute in a practical setting.

In flowmetric plethysmography with histamine bronchoprovocation at rest, circumferential changes in the body are measured using sensors and the airflow from the nostrils is measured using a face mask (pneumotachograph) [68]. The discrepancy between the airflow at the nose and the airflow at the chest represents the respiratory resistance [65]. This method is straightforward to perform, but it lacks the sensitivity required to detect minor limitations in lung function [69], due to its dependence on environmental factors [70].

Another method for determining respiratory resistance is impulse oscillometry [71]. In forced oscillatory mechanics, pulses are sent into the lungs and the return path is measured using sensors. In the presence of bronchoconstriction or a large accumulation of mucus due to EA, the return impulse is smaller, allowing for the assessment of respiratory resistance [65].

The above methods, particularly esophageal balloon pneumotachography, have a reduced sensitivity in diagnosing MEA compared to SEA [28]. In horses with mild symptoms, these techniques can be combined with histamine bronchoprovocation or bronchodilation with albuterol. Horses with MEA demonstrate bronchoconstriction at lower histamine doses than healthy horses. In horses with increased pulmonary resistance, bronchodilator challenge presents an opportunity to measure the capacity of the airways to bronchodilate [1,65].

Electrical impedance tomography (EIT) has been demonstrated to be an effective method for visualizing lung ventilation and perfusion without sedation or anesthesia [72,73]. Electrodes are placed around the equine thorax, which then generate a cross-sectional image of impedance changes based on changing ratios of air, fluid, fat, and ion concentration [74]. This allows for the detection of pathological changes such as pulmonary fibrosis and fluid accumulation [72]. While implementations for equine medicine are still under development, approaches are already available [64]. EIT enables the precise determination of peak inspiratory and expiratory flow, which enables the measurement of bronchoconstriction induced by exercise. This is observed to be enhanced in horses with severe equine asthma following an exercise test in comparison to healthy horses. Conversely, no significant correlation is evident in horses with mild asthma, which renders this method unsuitable for the early diagnosis of mild asthma [75].

Apart from exercise prior to pulmonary function testing, sedation with alpha 2-agonists also affects the outcomes of pulmonary function tests. Sedation with detomidine and butorphanol results in hypoventilation of the lungs as a consequence of reduced respiratory rate [55] and bronchoconstriction due to parasympathetic stimulation [73,76]. The lowering of the horse’s head during sedation causes upper airway obstruction due to nasal mucous membrane swelling [77]. Commonly used sedatives including xylazine, detomidine, and acepromazine have initial bronchoconstrictive effects followed by bronchodilation. Depending on the timing of the PFT, this may lead to the falsification of results [73,76].

In contrast to arterial blood gas analysis, in which both factors, exercise and sedation, have a negative influence on the results, PFT results are falsified by sedation, whereas exercise leads to more sensitive PFT diagnostics.

In general, diagnostics using PFT should always be combined with further diagnostics such as the BALF cytology examination, especially in mild-to-moderate cases [67]. PFT alone does not offer sufficient information to diagnose or grade EA severity, as the sensitivity in diagnosing MEA is reduced. The information in the current literature on the correlation between PFT and BALF cytology is contradictory, with some studies showing a significant correlation [18,69,78] while others show the opposite [79,80].

## 5. Effects of Short-Term Exercise on Lower Airways

Short-term exercise may be beneficial for diagnosing MEA, which has been shown in asymptomatic racehorses, of which 50% were diagnosed as equine asthmatics based on the total cell count in the BALF exceeding 530 cells/microliter [81]. The number of neutrophil and eosinophil granulocytes did not increase in all horses exhibiting an elevated total cell count in the BALF. In contrast, an increase in BALF neutrophils was found in healthy horses after exercise, whereas no significant effect was shown in asthmatic horses [82]. A comparison of the complete cytology of the BALF rather than just the neutrophils has not been published to our knowledge. Therefore, it remains unclear whether the exercise-induced alterations in BALF cytology are exclusive to MEA-positive horses or extend to healthy individuals as a pro-inflammatory reaction as well.

TA cytology is also affected by exercise, with the percentage of neutrophils rising in TAs by 24–87% compared to the pre-exercise state. This is presumably due to an increase in respiratory mechanics, expiratory flow, and mucociliary transport, which brings the cell ratios closer to the true cytologic composition of the lungs [83,84]. Exercise prior to testing can be used to enhance the diagnosis of subclinical cases, although BALF cytology is still preferable to TA cytology as it is clearly more representative [20].

In addition to its impact on cytology, exercise stress on the airways alters the composition of inflammatory mediators. In horses with SEA in exacerbation, the concentration of endothelin, a bronchoconstrictive and vasoactive peptide, is significantly higher in BALF and blood samples compared to healthy horses. Furthermore, the concentration of endothelin in the BALF of asthmatic horses increases following exercise, whereas healthy horses do not demonstrate a comparable increase. The precise relationship between this increase and the impairment of lung function and the cytology of BALF remains unclear [82].

Looking at other species, several other factors may be relevant for the horse. In men, asthmatics with exercise-induced bronchoconstriction (EIB) demonstrate an elevation in histamine and interleukin-8 (neutrophil chemotactic factor) in the blood following an exercise test on a treadmill [85]. Additionally, the concentration of high-sensitivity C-reactive protein (hs-CRP) in the blood serum is elevated following acute exercise in asthmatics with exercise-induced bronchoconstriction (EIB) [86]. Moreover, this physical exertion affects the airway epithelium, leading to an overexpression of cysteinyl leukotrienes [87], a relative underproduction of prostaglandin E2, and an increase in eosinophilia within the airways [88,89]. The cytology of induced sputum from individuals who develop exercise-induced bronchoconstriction demonstrates a higher concentration of epithelial cells [89].

Similar outcomes have been observed in healthy human subjects. An increase in the total cell count, leukocyte count, and epithelial cell count in the nasal lavage fluid was found after 180 min of exercise [90]. In response to physical stress, catecholamine and cortisol levels rise, which results in an increase in inflammatory mediators, including cytokines, leukotrienes, and prostaglandins from the lower airways [91]. Exercise-induced damage to the airway epithelium is attributed to osmotic changes and inadequate conditioning of the air. An increase in bronchial epithelial cells in the sputum following a half marathon serves as evidence of damage to the airway epithelium. Additionally, an increase in interleukin-8 has been observed in induced sputum [92]. In horses, an interleukin-8 increase after exercise is seen in blood serum [93].These findings collectively indicate the presence of an inflammatory response, characterized by increased epithelial permeability, resulting from epithelial injury. This, in turn, leads to a heightened release of inflammatory mediators, a larger volume of sputum, and an increased presence of granulocytes within the airways.

Further research is required to analyze the effect of exercise stress testing and to answer the question of whether the effect differs cytologically between healthy and MEA-affected horses, and may help to differentiate between these.

## 6. Effects of Long-Term Exercise on Lower Airways

Long-term exercise in terms of routine training has also been demonstrated to impact BALF, particularly in healthy racehorses. This does not affect the percentages of neutrophils, eosinophils, and mast cells, but rather the total cell count, erythrocytes, and hemosiderophages. This is an indicator of non-specific pneumonia of unknown origin and additionally indicates a correlation between the intensity and duration of exercise and the occurrence of exercise-induced pulmonary hemorrhage (EIPH) [94]. A greater proportion of erythrocytes and also neutrophil granulocytes can be observed in racehorses subjected to a high exercise load in comparison to those under a lower exercise load [95].

Conversely, other findings are inconsistent, indicating reduced total cell counts and neutrophil levels in horses examined over a 10-week training period [96]. It seems plausible that the lower airways adapt to prior-examination training. The total cell count yielded no statistically significant results [23]. In other studies, a lower proportion of mast cells, a higher total cell count, and a higher number of macrophages and lymphocytes were observed in small BALF volumes [97].

In humans, research on the incidence of exercise-induced asthma indicates that competitive swimmers have a higher risk of developing asthma due to chlorine exposure than tennis players. Inflammation of the airways with granulocytes, macrophages, and lymphocytes, as well as airway remodeling, have been demonstrated [91,98,99]. Regular running also increases the number of polymorphonuclear leukocytes (PMNs) in sputum, but there is no evidence of activation of these cells [100].

In mice, the bronchial epithelium shows a loss of ciliated cells after regular exercise, a slight increase in thickness, an unchanged presence of club cells (secretory active), increased apoptosis, and proliferation. The number of leukocytes in the walls and lumen of bronchioles is higher in exercised than in unexercised mice. However, these inflammatory cells do not appear to be activated [101].

This shows that the results regarding the effects of long-term exercise on the airways are not consistent. It is difficult to compare the literature, which consists of different training constructs and study designs. The theory of long-term exercise influencing the airways is understandable, but regarding the goal of the early diagnosis of mild EA, short-term exercise seems to be more appropriate.

## 7. Influence of Inhaling Cold Air on the Airways

In addition to short- and long-term exercise (training), respiratory stress due to environmental factors such as cold or chlorinated air has an impact on cytology. These factors trigger an inflammatory response in the lungs, because cold and dry air is not sufficiently warmed by the time it reaches the lungs [91]. In humans, this is known as “ski asthma” [102]. The resulting epithelial damage leads to the release of inflammatory mediators and neutrophilia in the airways, lung tissue remodeling, and bronchial hyperreactivity [103]. The damage is caused by the inhalation of cold air or, in swimmers, by the inhalation of chlorinated air [100,104].

In healthy horses, the inhalation of cold air during exercise results in an increase in bronchial epithelial cells and cytokines characteristic of the Th2 phenotype in BALF. The cytokines that are upregulated after cold air inhalation (−5 degrees) are mainly IL-4, IL-5, and IL-10, as well as IL-2 and IL-6 to a lesser extent. Activated mast cells are known to be involved in Th2 cytokine production, although other sources may be involved, as BALF contains very low levels of mast cells after cold air exercise. It has been suggested that lower respiratory tract lymphocytes amplify the cytokine response, while their production is preceded by other cells such as mast cells. A cellular inflammatory response could not be detected in BALF five hours after exercise, which may be due to the timing of BAL. This means that the inhalation of cold air promotes inflammatory processes in the lungs that appear to be similar to asthma [105]. As previous studies relate to healthy horses, future research concerning a cold air stress test on horses suffering from MEA would be beneficial. Compared to a standardized exercise test under natural environmental conditions, cold air exercise requires greater efforts and probably results in greater epithelial damage. It is necessary to evaluate the associated health risks and evaluate whether it provides an added value in terms of a more sensitive MEA diagnosis.

## 8. Co-Incidence of Pulmonary Hemosiderosis and Other Respiratory Diseases

As well as the aforementioned diagnostic influences, other diseases may precipitate the occurrence of EA or vice versa.

**Figure 2:**
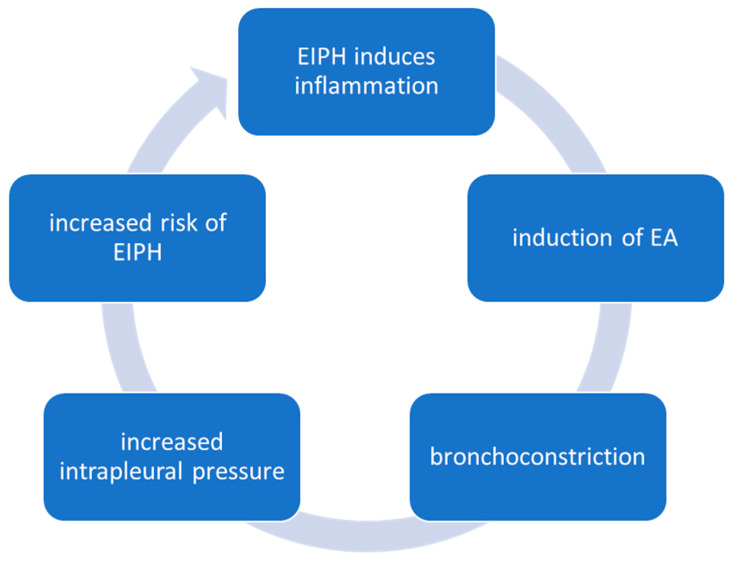
Pathomechanism of mutual influence of EIPH and EA.

Hemosiderophages can also be detected in the BALF of asthmatic horses, in addition to horses suffering from EIPH, especially racehorses, that are exposed to intense exercise. The quantity of hemosiderophages found in BALF is positively correlated with the severity of EA [106]. Leucocyte counts from BALF cytology in EA, EIPH, or combined disease show no significant differences. The inflammatory response appears to be similar, with one disease possibly causing the other. The pathomechanism of this mutual influence between EIPH and EA is based on bleeding in the lungs, which causes an inflammatory response. This inflammatory response can induce equine asthma (Figure 2). The resulting bronchoconstriction leads to an increased intrapleural pressure, which in turn increases the risk of EIPH [107]. Experimentally induced pneumonia also increases the risk of EIPH [108]. This is due to the altered vascular permeability caused by the inflammation. In the case of severe exercise, the increase in blood pressure is an important cause of EIPH. Angiogenesis and fibrosis induced by inflammation exacerbate the situation [109].

A correlation between respiratory disease and the occurrence of pulmonary hemosiderosis is also common in dogs and cats [110]. Hemosiderophages can be detected in 75% of tracheal lavage samples from cats suffering from feline asthma. Increased erythrocyte diapedesis is the result of increased pulmonary vascular congestion due to cytokine release and vasodilation. In addition, microtrauma of the pulmonary vessels due to coughing may be a contributing factor [111]. In humans, idiopathic pulmonary hemosiderosis is known to induce asthma-like episodes of bronchiolitis [112].

These findings in horses and other species suggest that it is important to consider the possible coexistence of EA and EIPH and that one condition may cause the other. Consequently, the etiology of EA may differ from the conventionally considered cause. This is of paramount importance for the management and treatment of the disease. When finding hemosiderophages in the BALF of non-racing asthmatic horses, it is recommended to investigate other potential causes of hemosiderophages in the lungs, beyond asthma and EIPH. Such a cause is atrial fibrillation [113]. Additionally, recurrent laryngeal neuropathy is considered a potential cause; however, a recent study conducted with a rather small and special population (Belgian horses and Percherons) was unable to establish a correlation [114]. In heavy horses and warmbloods, other causes may be more likely than intense exercise.

## 9. Conclusions

The respiratory tract reacts differently to various stresses. These stress factors influence a number of parameters commonly used in EA diagnostics, including aBGA, PFT, the scoring of respiratory secretion, and TA or BALF cytology. Transport, excitement, and sedation may negatively impact the clinical interpretation of aBGA. In contrast, the impact of exercise and cold air stress on cytology might be useful to amplify the diagnostic findings for an early diagnosis during mild and moderate disease. MEA diagnostics may be improved by a standardized exercise test before performing the bronchoalveolar lavage to differentiate between mild and moderate disease. The objective of further research is to evaluate whether exercise influences cytology differently in healthy and asthmatic horses, so that it can be used to identify subclinical cases of MEA or SEA in remission. It is of the utmost importance to diagnose EA early in the course of the disease and to define equine specific endotypes independently from phenotypes.

## Data Availability

Not applicable.
